# East Asian patients who received immunotherapy‐based therapy associated with improved survival benefit in advanced non‐small cell lung cancer: An updated meta‐analysis

**DOI:** 10.1002/cam4.7080

**Published:** 2024-03-08

**Authors:** Yueyuan Yao, Butuo Li, Yiyue Xu, Linlin Yang, Bing Zou, Linlin Wang

**Affiliations:** ^1^ Shandong First Medical University and Shandong Academy of Medical Sciences Jinan China; ^2^ Department of Radiation Oncology Shandong Cancer Hospital and Institute, Shandong First Medical University and Shandong Academy of Medical Sciences Jinan China

**Keywords:** chemotherapy, East Asia, immune checkpoint inhibitors, immunotherapy, meta‐analysis, non‐East Asia, non‐small cell lung cancer

## Abstract

**Background:**

Immune checkpoint inhibitors (ICIs) combined with chemotherapy have been recommended as the standard treatment for advanced NSCLC patients without driver‐gene mutations. However, there are different genetic characteristics and biological traits of tumors between non‐East Asian (nEA) and East Asian (EA) patients with NSCLC, which may contribute to differences in the efficacy of ICIs in different ethnic populations. Previous findings regarding differences in the efficacy of ICIs among ethnic groups have been inconsistent. Therefore, we performed a meta‐analysis by collecting published data to investigate the clinical outcomes of ICIs for EA NSCLC patients compared to nEA patients.

**Methods:**

Overall survival (OS) and progression‐free survival (PFS) were used to access the difference in survival outcomes between the two populations. Subgroup analyses were performed based on the line of ICIs, the use of ICIs alone or in combination, and the type of ICIs.

**Results:**

A total of 9826 NSCLC patients from 21 randomized controlled trials (RCTs) with 4064 EAs were included, which involved PD‐1, PD‐L1, and CTLA‐4 inhibitors. EA NSCLC patients who received ICIs‐based therapy were associated with significantly improved survival benefits in OS (*p* = 0.02) compared with nEA patients. Subgroup analysis indicated that EA patients receiving first‐line ICIs showed significantly superior OS compared with nEA patients (*p* = 0.007). Chemo‐ICIs treatment showed significant advantages in terms of OS (*p* = 0.002) and PFS (*p* = 0.02) among EA patients compared to nEA patients. In addition, PD‐1 inhibitors were associated with improved OS among both EA patients and nEA patients compared with PD‐L1 inhibitors.

**Conclusion:**

EA NSCLC patients who received ICIs‐based therapy were associated with significantly improved survival benefits compared with nEA NSCLC patients. Earlier intervention with ICIs and combination treatment was more recommended for EA NSCLC patients. Moreover, PD‐1 inhibitors are associated with prolonged survival among both EA and nEA patients.

## 1 INTRODUCTION

Lung cancer remains one of the most common cancer types all around the world, and non‐small cell lung cancer (NSCLC) accounts for most cases of lung cancer.^1^ Approximately 40%–70% of newly diagnosed patients are diagnosed at an advanced stage. Immune checkpoint inhibitors (ICIs) have shown tremendous benefit in the treatment of advanced NSCLC patients, with 5‐year survival increased from 5% to 30%.^2^ Thus, ICIs, including pembrolizumab, nivolumab, sintilimab, camrelizumab, etc., are recommended by National Comprehensive Cancer Network (NCCN) guidelines as standard strategies for advanced NSCLC patients.

More than 20% of drugs approved by the U.S. Food and Drug Administration (FDA) have reported racial differences in efficacy and safety.^3^ It should be noted that most trials of ICIs for NSCLC were initially conducted in Western countries, which enrolled only 3%–25.2% participants of EAs.^4^ However, there are different genetic characteristics and lifestyles between nEA and EA NSCLC patients, which leads to the variable clinical features and treatment recommendations.^5^, ^6^ The racial disparities in the efficacy of ICIs‐based therapy for advanced NSCLC are also of interest between EA and nEA patients.

While the direct comparison of the efficacy of ICIs in patients with advanced NSCLC between EA and nEA populations has not been conducted previously, existing findings of the outcomes of ICIs in EA population remain inconclusive. Longer survival was observed in the extension cohort of the Chinese subgroup in KEYNOTE‐042 study compared to the overall patient population, with median OS of 20.2 and 16.7 months, respectively.^7^, ^8^ Conversely, a meta‐analysis of 7 studies, including 5445 patients reported that there was no significant difference in the efficacy of ICIs between EA and nEA patients with NSCLC;^9^ however, the small number of studies and EA patients may limit the accuracy of the results. Additionally, given the differences of characteristics in EA versus nEA populations, the factors related to the efficacy of immunotherapy might differ, such as PD‐L1 expression, tumor mutation burden, genic mutation, etc.^10^, ^11^, ^12^, ^13^ Therefore, the impact of the above factors should also be taken into consideration when comparing the efficacy between the two populations.

## 2 MATERIALS AND METHODS

### 2.1 Search strategy and Study Selection criteria

This meta‐analysis was conducted following the Preferred Reporting Items for Systematic Reviews and Meta‐Analyses (PRISMA) guidelines. Study selection and data extraction were independently performed by two investigators (Yueyuan Yao and Butuo Li). Differences were determined by a third investigator (Linlin Wang) and resolved by consensus. PubMed, Embase, and the Cochrane Library were explored to identify works published from January 2015 to April 2023. Furthermore, relevant abstracts presented at major conferences, including the American Society of Clinical Oncology (ASCO), American Society for Radiation Oncology (ASTRO), American Association for Cancer Research (AACR), European Lung Cancer Conference (ELCC), European Society for Medical Oncology (ESMO), World Conference on Lung Cancer (WCLC), and Chinese Society of Clinical Oncology (CSCO), were also reviewed to identify unpublished studies and updated outcomes.

Phase II/III prospective RCTs that reported the efficacy of ICIs‐alone or ICIs‐Chemo combined for advanced NSCLC patients were included. The following keywords were used in our search strategy: non‐small‐cell lung cancer, NSCLC, immunotherapy, ICIs, programmed death receptor 1, PD‐1, programmed death‐ligand 1, PD‐L1, cytotoxic T lymphocyte‐associated antigen‐4, CTLA‐4, RCT, randomized controlled trial. Studies without available survival data on EA or nEA populations were excluded by manual scanning.

### 2.2 Data extraction and quality assessment

The following data were extracted from each included study: the name of RCTs, first author, publication year, publication type, NCT number, phase of trials, number of patients, tumor histology, ethnicity, stage of NSCLC, line of treatment, type of ICIs, name of ICIs, and original geographical regional classification. In addition, the hazard ratio (HR) estimated and 95% confidence intervals (CI) for time to outcomes (OS and PFS) in EA or nEA were also retrieved from the related literature, respectively.

### 2.3 Statistical analysis

The primary endpoints of interest were outcomes in different ethnical patients, including OS and PFS, to systemically evaluate the difference in efficacy of ICIs between the EA and nEA populations. HR and their corresponding 95% CI were estimated using fixed/random effect model. Subgroup analyses were performed to explore the potential risk factors for patients in different groups. The potential for publication bias was shown in funnel plots. Heterogeneity was evaluated by the *χ* ^*2*^ and *I* ^2^ test, and a *p*‐value < 0.1 of the *χ* ^2^ or *I* ^2^ ≥ 50% was considered to indicate statistically significant heterogeneity. The random‐effects model was used if heterogeneity was presented; otherwise, the data were analyzed by the fixed‐effects model. All analyses were performed with Review Manager (RevMan) 5.4.1.

## 3 RESULTS

### 3.1 Eligible studies and characteristics

**FIGURE 1:**
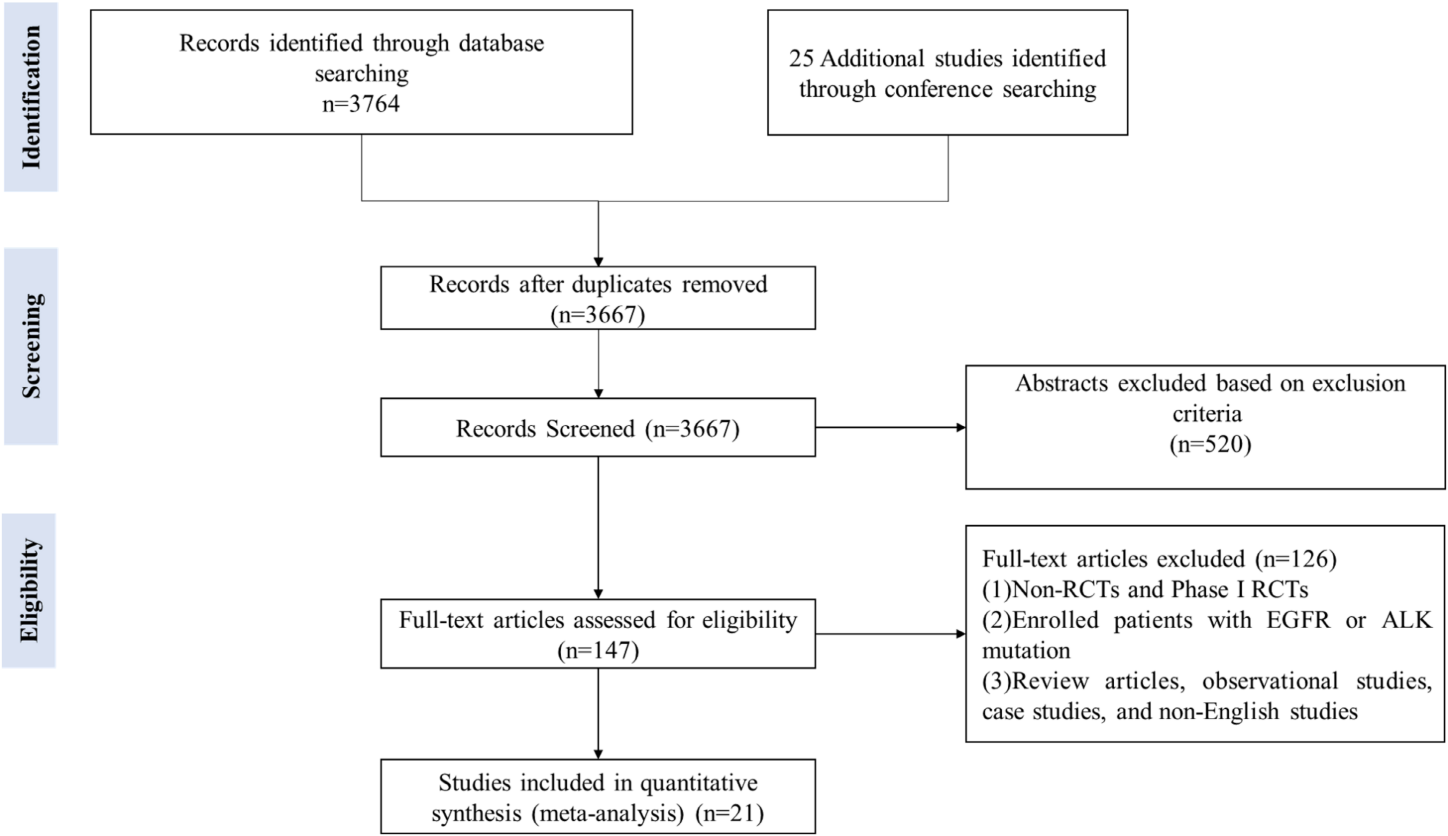
PRISMA flow diagram for the meta‐analysis.

We retrieved 3789 references initially. After carefully screening the abstracts and eliminating the duplicates, 147 studies were considered eligible for full‐text review, and 21 RCTs were finally included. The selection steps and eligibility criteria were summarized in the flow diagram (Figure 1).

A total of 9826 NSCLC patients from 21 studies were enrolled in this meta‐analysis, of which 4064 (42%) were EAs in 14 studies and 5762 (58%) were nEAs. The major characteristics at baseline of the included studies are presented in Table 1. All included studies were phase III RCTs, except for KEYNOTE‐010 (phase II/III). Five studies focused on PD‐L1 inhibitors, 14 studies focused on PD‐1 inhibitors, and the remaining two focused on the combination of PD‐1 and CLAT‐4 inhibitors. Patients from control group received chemotherapy in all studies. On the other hand, 13 trials compared the efficacy of ICIs‐Chemo with chemotherapy, six trials compared the ICIs alone against chemotherapy, and two trials compared CTLA‐4 plus PD‐1 inhibitor (IPI + NIVO) against chemotherapy. ICIs were used as first‐line treatment in 17 studies, and the rest four studies involved second‐ or beyond‐line application of ICIs.

**TABLE 1:** Main study characteristics of the eligible randomized controlled trials.

Study	Author	Year	NCT	Phase	Histology	Stage	Sample Size	Line	Comparison Groups	Original Subgroups	Group	Number of patients
IMpower132^17^, ^31^	Lu et al.	2021	NCT02657434	III	nSq‐NSCLC	IV	163	1	Atezolizumab+Chemo versus Chemo	China	East Asia	82
	Nishio et al.	2021	NCT02657434	III	nSq‐NSCLC	IV	578	1		Asia	East Asia	71
										White	non‐East Asia	193
IMpower150^32^	Socinski et al.	2021	NCT02366143	III	nSq‐NSCLC	IV	1202	1	Atezolizumab+Chemo versus Chemo	Asia	East Asia	27
										White	non‐East Asia	300
KN‐024^15^, ^33^, ^34^	Reck et al.	2016	NCT02142738	III	NSCLC	IV	305	1	Pembrolizumab versus Chemo	East Asia	East Asia	40
		2019	NCT02142738	III	NSCLC	IV	305	1		non‐East Asia	non‐East Asia	265
	Satouchi et al.	2020	NCT02142738	III	NSCLC	IV	40	1		Japan	East Asia	21
KN‐407^35^, ^36^	Paz‐Ares et al.	2018	NCT03875092	III	Sq‐NSCLC	IV	549	1	Pembrolizumab+Chemo versus Chemo	East of Asia	East Asia	52
			NCT03875092	III	Sq‐NSCLC	IV	549	1		Rest of the world	non‐East Asia	224
	Cheng et al.	2021	NCT03875092	III	Sq‐NSCLC	IV	125	1	Pembrolizumab+Chemo versus Chemo	China	East Asia	65
KN‐010^37^	Herbst et al.	2021	NCT01905657	II/III	NSCLC (PD‐L1 ≥1%)	IIIB/IV	1034	>1	Pembrolizumab versus Chemo	East Asia	East Asia	62
										non‐East Asia	non‐East Asia	281
KN‐042^8^	Mok et al.	2019	NCT02220894	III	NSCLC	Locally advanced/Metastatic	1374	1	Pembrolizumab versus Chemo	East Asia Europe Latin American other	East Asia non‐East Asia	185
												452
KN042 China^7^	Wu et al.	2020	NCT03850444	III	NSCLC (PD‐L1 ≥1%)	Locally advanced/Metastatic	264	1	Pembrolizumab versus Chemo	China	East Asia	128
JAVELIN Lung 200^38^, ^39^	Barlesi et al.	2018	NCT02395172	III	NSCLC	IIIB/IV/Recurrent	792	>1	Avelumab vrsus Chemo	Asia USA and western Europe Eastern Europe Rest of the world	East Asia non‐East Asia	69
	Park et al.	2021	NCT02395172	III	NSCLC		792	>1				495
CM‐078^40^	Lu et al.	2021	NCT02613507	III	NSCLC	IIIB/IV/Recurrent	504	>1	Nivolumab versus Chemo	China	East Asia	302
										non‐China	non‐East Asia	36
CM‐227^41^, ^42^	Hellmann et al.	2018	NCT02477826	III	NSCLC with high TMB	IV/Recurrent	299	1	Nivolumab+Ipilimumab versus Chemo	Asia North America Europe Rest of world	East Asia non‐East Asia	21 118
	Byrne et al.	2022	NCT02477826	III	NSCLC	IV/Recurrent	245	1	Nivolumab+Ipilimumab versus Chemo	Asia	East Asia	121
CM‐9LA^51^	John et al.	2022	NCT03215706	III	NSCLC	IV/Recurrent	58	1	Nivolumab+Ipilimumab+Chemo vs. Chemo	Asia	East Asia	28
MYSTIC^43^	Rizvi et al.	2020	NCT02453282	III	NSCLC	IV	1891	1	Durvalumab versus Chemo	Asia non‐Asia	East Asia non‐East Asia	59 103
KN‐189^44^	Horinouchi et al.	2021	NCT03950674	III	nSq‐NSCLC	IV	40	1	Pembrolizumab+Chemo versus Chemo	Japan	East Asia	25
ORIENT‐11^45^	Yang et al.	2021	NCT03607539	III	nSq‐NSCLC	Locally advanced/Metastatic	397	1	Sintilimab+Chemo versus Chemo	China	East Asia	266
ORIENT‐12^46^	Zhou et al.	2021	NCT03629925	III	Sq‐NSCLC	Locally advanced/Metastatic	357	1	sintilimab+Chemo versus Chemo	China	East Asia	179
GEMSTONE‐302^52^	Zhou et al.	2022	NCT03789604	III	NSCLC	IV	479	1	Sugemalimab+Chemo versus Chemo	China	East Asia	320
CameL‐nSq^53^	Zhou et al.	2020	NCT03134872	III	nSq‐NSCLC	IIIB/IV	419	1	Camrelizumab+Chemo versus Chemo	China	East Asia	205
CameL‐Sq^47^	Sheng et al.	2022	NCT03668496	III	Sq‐NSCLC	IIIB/IV	389	1	Camrelizumab+Chemo versus Chemo	China	East Asia	193
RATIONALE 307^48^	Wang et al.	2021	NCT03594747	III	Sq‐NSCLC	IIIB/IV	355	1	Tislelizumab+Chemo versus Chemo	China	East Asia	119
RATIONALE 304^49^	Lu et al.	2021	NCT03663205	III	nSq‐NSCLC	IIIB/IV	334	1	Tislelizumab+Chemo versus Chemo	China	East Asia	223
CHOICE‐01^50^	Wang et al.	2021	NCT03856411	III	NSCLC	IIIB/IV	465	1	Toripalimab+Chemo versus Chemo	China	East Asia	309

### 3.2 Outcome differences between EA versus nEA

**FIGURE 2:**
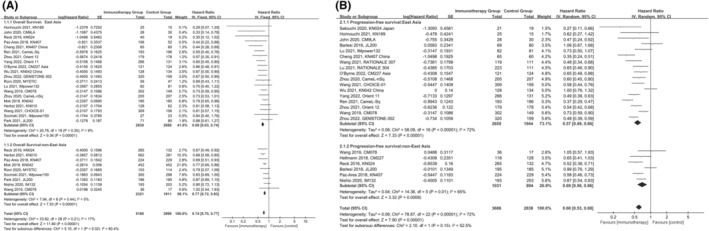
Comparison of hazards ratio of OS (A) and PFS (B) between East Asian and non‐East Asian patients.

The HR for OS of each study and the results of pooled HR based on the fixed‐effects models are summarized in Figure 2A. The overall estimated fixed‐effects HR for OS is 0.74 (95% CI 0.70–0.77), with a statistically significant 26% reduction in the hazard of death with ICIs‐based therapy compared with chemotherapy. Among patients from East Asia, ICIs‐based therapy could reduce the risk of death by 31%, and the pooled HR for OS was 0.69 (95% CI 0.63–0.74) with low heterogeneity (*I* ^2^ = 9.0%, *p* = 0.35). A similar superiority was also observed in nEA patients with a decrease in the risk of death by 23% (HR: 0.77, 95% CI 0.72–0.83) without statistical heterogeneity (*I* ^2^ = 0%, *p* = 0.44). Interestingly, the advantage of ICIs was more significant in EAs compared to nEAs (*p* = 0.02).

The HR of PFS of the individual studies and the combined results according to the random‐effects models are summarized in Figure 2B. The overall estimated random‐effects HR for PFS is 0.60 (95% CI 0.53–0.68), with a statistically significant 40% reduction in the hazard of PFS in the treatment group compared to the control group. Consistent with OS, the meta‐analysis also showed that ICIs‐based therapy could notably prolong PFS in patients with NSCLC from whether East Asia (HR: 0.57, 95%CI 0.49–0.66, *p* < 0.00001) or non‐East Asia (HR: 0.69, 95% CI 0.56–0.86, *p* = 0.0009). Although the benefit of ICIs was obvious among patients from East Asia compared to non‐East Asia, no statistical significance was observed (*p* = 0.15). While, there was statistical heterogeneity in both EA group (*I* ^2^ = 72%, *p* < 0.00001) and nEA group (*I* ^2^ = 65%, *p* = 0.01).

Considering the important role of the basal expression of PD‐L1, the comparative analysis of outcomes between EAs and nEAs was also performed for patients with different PD‐L1 expression. In terms of patients with PD‐L1 ≥ 1%, the superior benefit of OS was observed in EA patients compared to nEA patients (*p* = 0.04; Figure S3A). However, no statistical difference in PFS was observed between the two groups in EA patients (Figure S3B). However, the comparison of patients without basal expression of PD‐L1 (<1%) between EA and nEA patients was not performed due to the limited data.

### 3.3 Subgroup analysis

#### 3.3.1 Treatment lines

**FIGURE 3:**
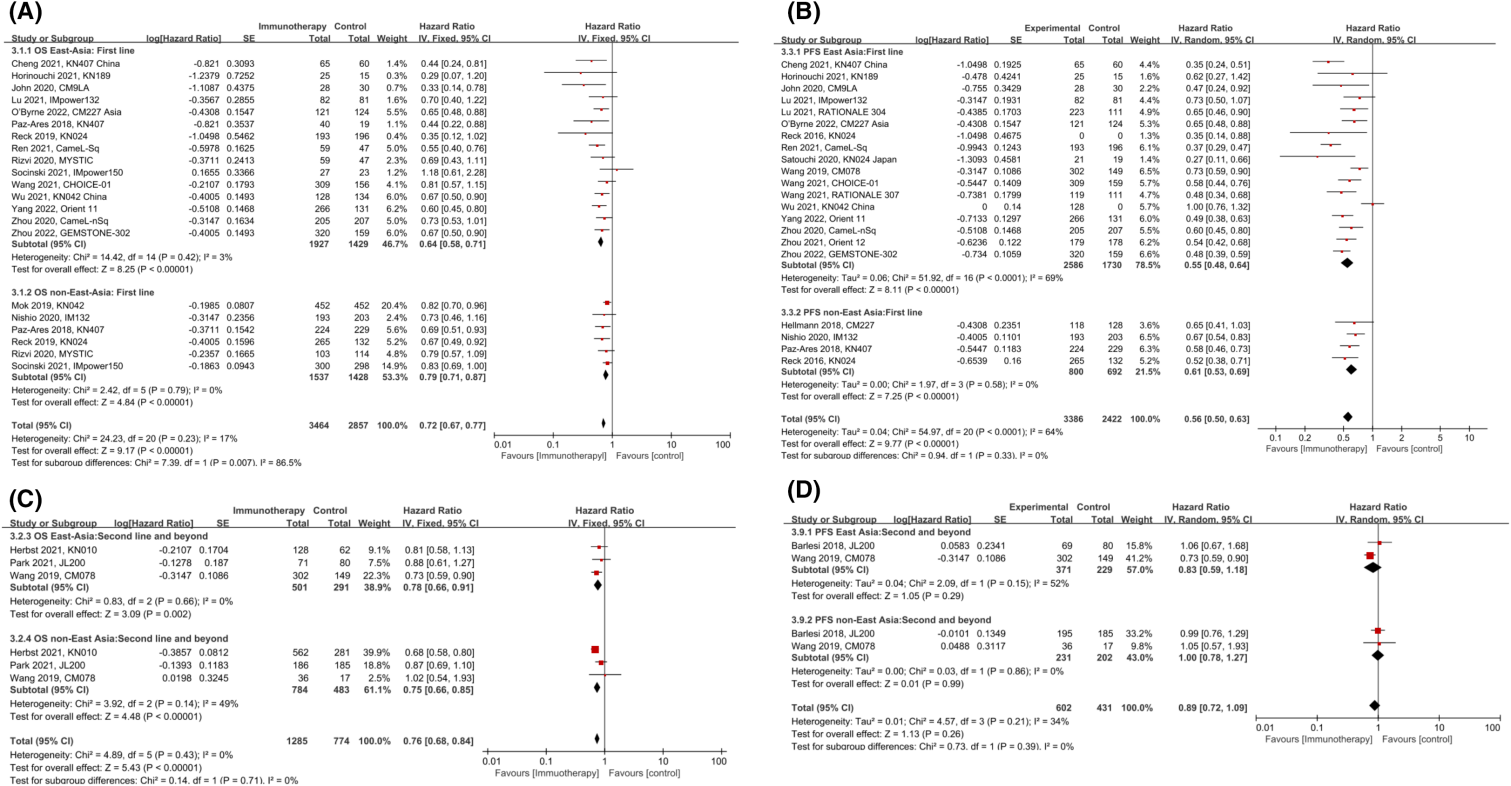
Subgroup comparison of hazards ratio of survival according to treatment line. Comparison of hazards ratio of OS (A) and PFS (B) between East Asian and non‐East Asian patients in the first line treatment group. Comparison of hazards ratio of OS (C) and PFS (D) between East Asian and non‐East Asian patients in the second and beyond treatment group.

Of the 21 trials included in this analysis, 16 evaluated the efficacy of ICIs in the first‐line treatment. As shown in Figure 3A, improved OS from first‐line ICIs was shown in both EA (HR: 0.64, 95% CI 0.58–0.71, *p* < 0.00001) and nEA patients (HR: 0.79, 95% CI 0.71–0.87, *p* < 0.00001). Compared with nEA NSCLC patients, significant improvement in OS was shown in EA patients with a reduction of 28% in the risk of death (*p* = 0.007). Similarly, superiority was also shown in PFS for both EAs (HR: 0.55, 95% CI 0.48–0.64, *p* < 0.0001) and nEAs (HR: 0.61, 95% CI 0.53–0.69, *p* < 0.00001). However, the PFS benefit of first‐line ICIs was similar in EA and nEA patients as shown in Figure 3B (*p* = 0.33).

As shown in Figure 3C,D, the significant efficacy benefits from subsequent line ICIs were shown in OS but not in PFS, both in EA and nEA patients. And no significant difference was observed between the two populations. However, these findings might be influenced by the relatively small sample size and the number of included trials.

#### 3.3.2 Combination with chemotherapy or not

**FIGURE 4:**
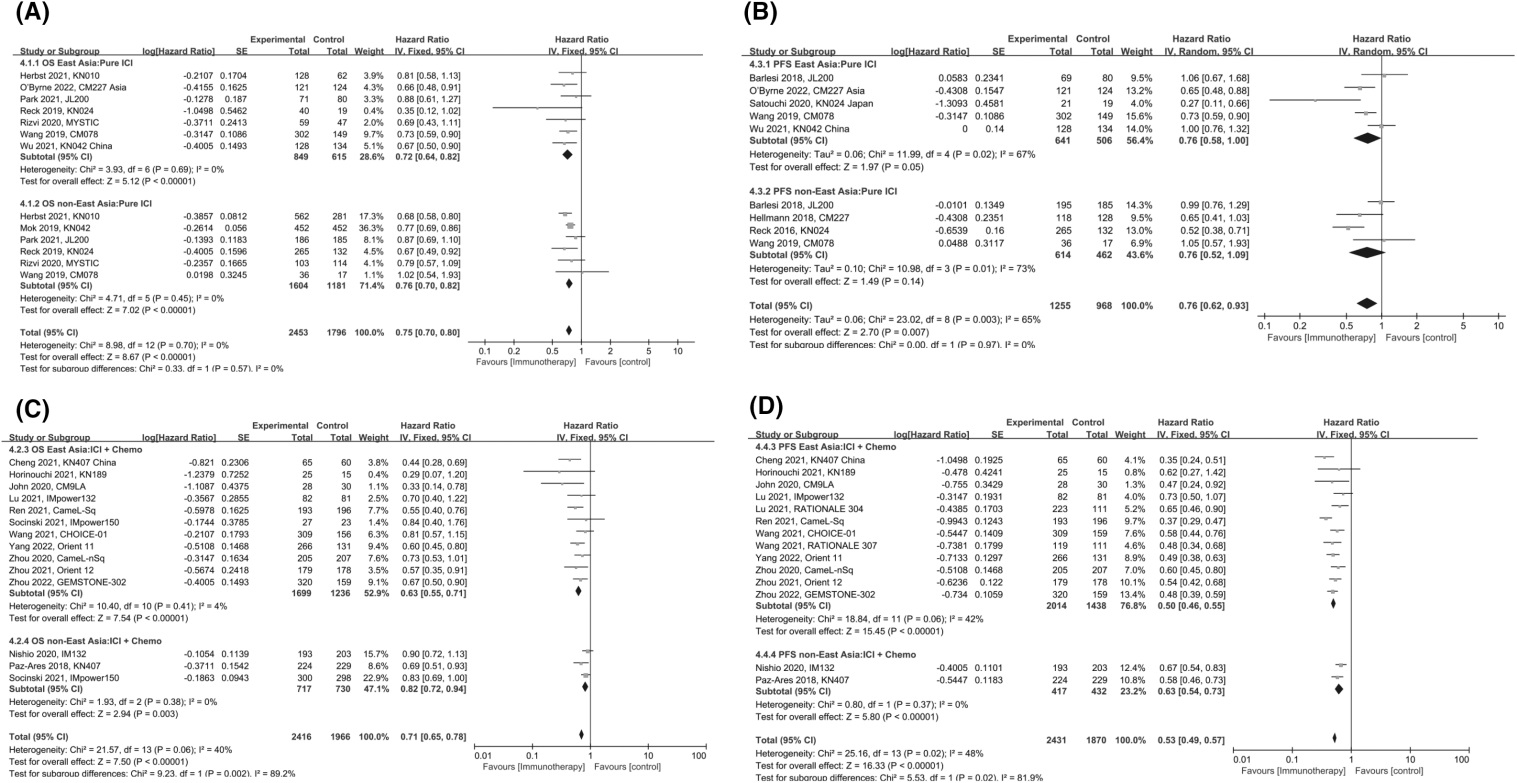
Subgroup comparison of hazards ratio of survival according to combination treatment or not. Comparison of hazards ratio of OS (A) and PFS (B) between East Asian and non‐East Asian patients in the pure ICIs treatment group. Comparison of hazards ratio of OS (C) and PFS (D) between East Asian and non‐East Asian patients in the combination treatment of ICIs and immunotherapy.

Compared to chemotherapy, ICIs alone were associated with improved OS (HR: 0.75, 95% CI 0.70–0.80, *p* < 0.00001) and PFS (HR: 0.76, 95% CI 0.62–0.93, *p* = 0.007) in advanced NSCLC patients. The improved OS (Figure 4A) was observed both in EA (HR: 0.72, 95% CI 0.64–0.82, *p* < 0.00001) versus nEA populations (HR: 0.76, 95%CI 0.70–0.82, *p* < 0.00001), but there was no statistical difference between the two groups (*p* = 0.57).

In terms of PFS (Figure 4B), significant improvement was only observed in EAs (HR: 0.76, 95% CI 0.58–1, *p* = 0.05) but not in nEA patients (HR: 0.76, 95% CI 0.52–1.09, *p* = 0.14). Consistent with OS, the PFS benefit of ICI monotherapy was also not statistically different between the two populations (*p* = 0.97). As shown in Figure 4C,D, ICI‐Chemotherapy treatment was also associated with the improvement of OS and PFS in both EA and nEA population. Compared to nEAs, there was statistically significant prolonged OS (*p* = 0.002) and PFS (*p* = 0.02) in EA population.

To explore a better treatment strategy for patients of different races, we further compared the efficacy of ICIs‐alone and ICIs‐Chemo combined in the two populations, respectively. For EA patients, significant improvement in PFS was observed in the combination treatment group (*p* < 0.00001). Prolonged OS was also observed, but with no statistical difference compared to ICI alone (*p* = 0.10) (Figure S1A,B). For nEA patients, no survival benefit from combination treatment was observed, whether in PFS (*p* = 0.53) or OS (*p* = 0.09) (Figure S1C,D).

#### 3.3.3 Type of ICIs

**FIGURE 5:**
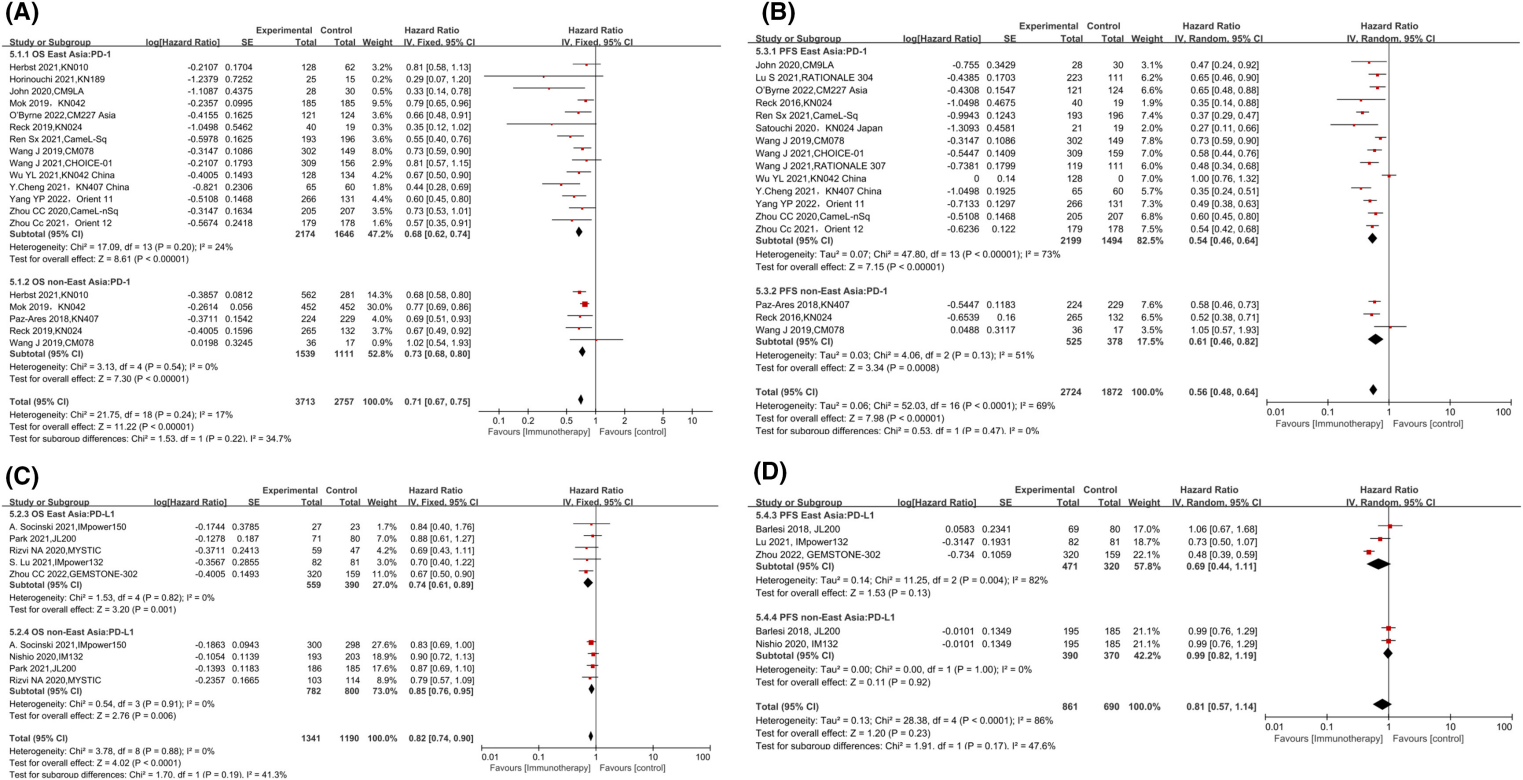
Subgroup comparison of hazards ratio of survival according to type of ICIs. Comparison of hazards ratio of OS (A) and PFS (B) between East Asian and non‐East Asian patients receiving PD‐1 inhibitors. Comparison of hazards ratio of OS (C) and PFS (D) between East Asian and non‐East Asian patients receiving PD‐L1 inhibitors.

In terms of PD‐1 inhibitors, EA patients had a 32% decrease in the risk of death with the pooled HR of 0.68 (95% CI: 0.62–0.74) for OS (Figure 5A). The similar superiority was also seen in nEA patients with a decrease in the risk of death by 27% (HR: 0.73, 95% CI 0.68–0.80, *p* < 0.00001). As shown in Figure 5B, significant PFS benefits from PD‐1 inhibitors were also observed whether in EAs (HR: 0.54, 95% CI 0.46–0.64, *p* < 0.00001) or nEAs (HR: 0.61, 95% CI 0.46–0.82, *p* = 0.0008). Whereas, there was no statistically significant difference between EA and nEA populations on OS (*p* = 0.22) and PFS (*p* = 0.47) benefit from PD‐1 inhibitors.

As shown in Figure 5C,D, survival benefit from PD‐L1 inhibitors was only observed in OS (HR: 0.82, 95% CI 0.74–0.90, *p* < 0.0001) but not in PFS (HR: 0.81, 95% CI 0.57–1.14, *p* = 0.23). Similar survival benefits were observed in both East and nEA patients, with no significant difference in OS (*p* = 0.19) and PFS (*p* = 0.17) between the two populations.

We further compared the efficacy of PD‐1 inhibitors versus PD‐L1 inhibitors in EA and nEA populations, respectively, to identify better treatment strategies for different populations. The results indicated that compared to PD‐L1 inhibitors, PD‐1 inhibitors exhibited superior OS (*p* = 0.04) and PFS (*p* = 0.006) in nEA populations (Figure 2C,D). For EA patients, PD‐1 inhibitors were only associated with prolonged OS (*p* = 0.02) but not PFS (*p* = 0.33; Figure 2A,B).

## 4 DISCUSSION

PD‐1/PD‐L1 inhibitors combined used with chemotherapy have been recommended as the standard treatment strategy by NCCN guidelines for advanced NSCLC patients without driver‐gene mutations according to the results of KEYNOTE‐024.^14^, ^15^ Notably, most clinical trials of ICIs were initially conducted in Western countries, where the majority of including patients were Caucasian.^16^ The portion of EA patients is also limited in multi‐ethnic studies, and the ethnic disparities have not been evaluated in the intent‐to‐treat cohort. Due to the differences in genetic, biological, and clinical characteristics between EA and nEA patients, the different roles of ICIs in different sub‐populations should be noted.

The different efficacy of ICIs between different ethnic populations has been explored before, but the results have been inconsistent. Subgroup analysis of IMpower132 indicated the superior PFS of atezolizumab in Asian NSCLC patients compared to white patients (HR: 0.42 vs. 0.67).^17^ However, the real‐world studies indicated similar efficacy from ICIs for Indian and Canadian patients with median OS of 11 and 12 months, respectively.^18^, ^19^ While direct comparison of numerical data from different studies is inappropriate due to different confounding factors between studies, a systemic review is needed.

The racial disparities of ICIs have also been systemically reviewed before, but no uniform conclusion was retrieved. A meta‐analysis, including 19 studies and 7 of them performed in NSCLC patients, compared the efficacy of ICIs in Asian versus non‐Asian cancer patients, and the results indicated significantly improved survival benefits in Asian patients receiving ICIs‐based thearpy.^20^ Peng et al. performed a meta‐analysis to compare the efficacy of ICIs in EA versus nEA patients with NSCLC, and found no statistical difference in response to ICIs between the two populations.^9^ However, only 7 studies of 5445 NSCLC patients were included in this meta‐analysis, of which 1740 patients were from East Asia. Besides, only two studies have reported the efficacy of PD‐L1 inhibitors in NSCLC patients. Currently, the available data on the efficacy of ICIs in EA patients has increased as more studies have been conducted in this sub‐population. Thus, we further updated the content of the review on the racial disparities of ICIs.

Our study included 21 phase II/III clinical trials with 4064 EA patients from 14 studies. Besides, the studies of additional new PD‐1 inhibitors (sintilimab, camrelizumab, tislelizumab, and toripalimab) and PD‐L1 inhibitors (sugemalimab and durvalumab) were included in analyses. Our results indicated the benefit from ICIs of advanced NSCLC patients, whether in EA or nEA, compared with chemotherapy. More importantly, better performance of ICIs was also observed in EA NSCLC patients compared to non‐Asian patients, both in OS and PFS. Based on the results of subgroup analyses, significant benefit from first‐line or combination treatment of ICIs was observed in East‐Asian NSCLC patients, which might be the reason for racial disparities in the efficacy of ICIs.

Although the mechanisms by which EA populations may benefit more from ICIs are still unclear, the different genomic profiles and lifestyle factors were found to be attributed to the different outcomes between the two sub‐populations. Patients in the nEA population were found to have a higher prevalence of smoking,^21^ which has several implications on tumor microenvironment (TME) such as the defect on immunologic homeostasis. And exposure to tobacco was also found to be associated with inferior outcomes in patients receiving immunotherapy.^22^ It should be noted that there is a significant difference in the genomic profiles between EA and non‐EA patients. In this analysis, 66.8% of nEA patients were Caucasian. Caucasian patients were found to have a higher prevalence of STK11 mutations, and Asian patients had a higher prevalence of EGFR mutations.^23^, ^24^ The ancillary analysis of the POPLAR and OAK studies also indicated the significant differences of genetic alteration profiles between Asian and white patients, including TP53, EGFR, STK11, KEAP1, KRAS, and others, which were found to be significantly associated with the survival of patients receiving immunotherapy. Notably, patients with EGFR mutations were mostly excluded from the studies of ICIs. It has been reported that genetic mutations affect the response to immunotherapy by modifying TME.^25^, ^26^, ^27^ Patients with STK11 alterations favored the TME with higher density of neutrophils, lower density of CD8^+^T cells in the stroma, and lower expression of PD‐L1, which might result in less efficacy from ICIs.^26^ Besides, nEA NSCLC was also associated with high genomic instability, high proliferation profile, and low immune infiltration,^4^, ^27^, ^28^ which could be associated with poor prognosis. The underlying mechanism of the difference in outcomes between ethnic patients’ needs further investigation.

Interestingly, different results of comparison were shown between the two populations in the ICIs monotherapy or ICIs combination therapy subgroups. The similar benefit was shown between the two populations receiving ICI monotherapy. However, favorable PFS and OS were observed in EA patients receiving ICIs‐Chemo combined therapy compared to nEAs. Previous studies have found that ICIs combination treatment is associated with significant benefit in PFS but not OS compared to ICIs alone.^28^ We found prolonged PFS and OS from ICIs combination treatment in EA patients but not in nEA patients. Thus, under the circumstances of unknown PD‐L1 expression, combination ICIs strategies are more recommended for East‐Asian patients.

In terms of variety of ICIs, both EA and nEA patients could benefit from PD‐1 inhibitors, and no significant difference was observed between the two populations. However, patients receiving PD‐1 inhibitors were indicated to obtain better OS rather than PFS compared to chemotherapy in both populations. Previous studies have shown the superior efficacy of PD‐1 inhibitors over PD‐L1 inhibitors in various tumor types.^29^ One possible reason is that PD‐1 inhibitors, which bind to PD‐1 in immune cells, can inhibit the binding of PD‐1 to both PD‐L1 and PD‐L2 completely. Whereas PD‐L1 inhibitors can only block the binding of PD‐1 to PD‐L1, which might in turn affect the complete activation of immune cells.^29^ It has also been reported that NSCLC patients exhibit higher PD‐L2 expression, which supports our observation that PD‐1 inhibitors have superior efficacy over PD‐L1 inhibitors in NSCLC patients.^30^ We further performed a subgroup analysis to identify whether PD‐1 and PD‐L1 inhibitors exhibited similar efficacy in EA and nEA NSCLC patients and found the superior efficacy of PD‐1 inhibitors both in OS and PFS in nEAs. However, the advantage of PD‐1 inhibitors for EAs was only observed in OS but not in PFS. Thus, PD‐1 inhibitors rather than PD‐L1 inhibitors should be first recommended for both populations.

This study also has several limitations. First, pooled data rather than individual patients was used for analysis because differences in baseline characteristics and other pooled data of trials could not be adjusted. Multiple Cox regression analyses could not be performed to analyze the predictive effect of various biological or demographic confounders, such as histological subtype, PD‐L1 expression, gender, and smoking status. Second, the results could not be obtained from clinical trials that didn't meet the primary endpoints, and only published trials were included in the analysis, which may introduce publication bias. Finally, survival outcome continues to be followed up in some inclusion trials, such as CameL, RATIONALE‐304, KEYNOTE‐024, and CheckMate‐9LA studies etc., where heterogeneity and risk of bias have their origins.

## 5 CONCLUSION

In summary, this study suggested that EA patients have better survival benefits than nEAs. For EA NSCLC patients, the earlier intervention of ICIs might lead to longer survival, and ICI combination treatment is more recommended. PD‐1 inhibitors were associated with prolonged survival from ICIs for both EA and nEA NSCLC patients.

## AUTHOR CONTRIBUTIONS

**Yueyuan Yao:** Data curation (equal); formal analysis (equal); investigation (equal); methodology (equal); writing – original draft (equal); writing – review and editing (equal). **Butuo Li:** Conceptualization (equal); formal analysis (equal); methodology (equal); writing – original draft (equal); writing – review and editing (equal). **Yiyue Xu:** Data curation (equal); investigation (equal); software (equal). **Linlin Yang:** Formal analysis (equal); software (equal). **Bing Zou:** Data curation (equal); formal analysis (equal); software (equal). **Linlin Wang:** Conceptualization (equal); supervision (equal).

## FUNDING INFORMATION

This research was supported by National Natural Science Foundation of China (Grant number 82172865), Start‐up fund of Shandong Cancer Hospital (Grant number 2020‐B14), Clinical Research Special Fund of Wu Jieping Medical Foundation (Grant number 320.6750.2021‐02‐51 and 320.6750.2021‐17‐13).

## CONFLICT OF INTEREST STATEMENT

All authors disclosed no relevant relationship.

## Supporting information


Figures S1–S4.

## Data Availability

The data that support the findings of this study are available from the corresponding author upon reasonable request.
