# Supplementary material for: East Asian patients who received immunotherapy‐based therapy associated with improved survival benefit in advanced non‐small cell lung cancer: An updated meta‐analysis
Source: Cancer Med. 2024 Mar 8;13(4):e7080. doi: 10.1002/cam4.7080 (PMC10923033; doi:10.1002/cam4.7080)
Supplement: Supplementary file 1 — Figures S1–S4. [file CAM4-13-e7080-s001.docx]

**Supplementary**

**

**

**Figure S1** Subgroup analysis of hazards ratio of survival between combined immunotherapy and immunotherapy alone in different racial population. Comparison of hazards ratio of OS (A) and PFS (B) between combined immunotherapy and immunotherapy alone in the group of East Asian patients. Comparison of hazards ratio of OS (C) and PFS (D) between combined immunotherapy and immunotherapy alone in the group of non-East Asian patients.





**Figure S2** Subgroup analysis of hazards ratio of survival between PD-1 inhibitors and PD-L1 inhibitors in different racial population. Comparison of hazards ratio of OS (A) and PFS (B) between PD-1 inhibitors and PD-L1 inhibitors in the group of East Asian patients. Comparison of hazards ratio of OS (A) and PFS (B) between PD-1 inhibitors and PD-L1 inhibitors in the group of non-East Asian patients.





**Figure S3** Subgroup analysis of hazards ratio of survival according with expression of PD-L1. Comparison of hazards ratio of OS (A) and PFS (B) between East Asian and non-East Asian NSCLC patients with the PD-L1 ≥ 1%





**Figure S4** Publication bias (funnel plot): OS(A) and PFS(B) in East Asia and non-East Asia.

**Funding**

This research was supported by Natural Science Foundation of Shandong Province, (Grant number ZR2019LZL012), Jinan Clinical Medical Science and Technology Innovation Plan (Grant number 202019043), National Natural Science Foundation of China (Grant number 8217102892), The Key Research and Development Program of Shandong (Major Science & Technology Innovation Project) (2021SFGC0501) and Start-up fund of Shandong Cancer Hospital (2020-B14).
